# Investigating the association between GLP-1 receptor agonists and mood disorders: A study integrating real-world data and Mendelian randomization

**DOI:** 10.1192/j.eurpsy.2025.10125

**Published:** 2025-12-03

**Authors:** Xiulan Zheng, Hao Wang, Ping Liu, Jie Pan, Rundong Lv, Chen Feng

**Affiliations:** 1 School of Pharmacy, Faculty of Medicine, Macau University of Science and Technology, Macau SAR, China; 2 Department of Urology, Zibo Central Hospital, Zibo, China; 3 Department of Clinical Pharmacy, Zibo Central Hospital, Zibo, China; 4 Department of Pharmacy, The Second Affiliated Hospital of Soochow University, Suzhou, China

**Keywords:** FAERS database, glucagon-like peptide-1 receptor agonists, Mendelian randomization, mood disorders

## Abstract

**Background:**

As GLP-1 receptor agonists (GLP-1 RAs) are increasingly used worldwide, concerns about their association with mood disorders have grown. Yet real-world observational studies have produced conflicting findings. This study aims to fully examine the link between GLP-1 RAs and emotional/behavioral outcomes.

**Methods:**

Disproportionality analysis of GLP-1 RA adverse events was conducted using FAERS data. Mendelian randomization (MR) employed GLP1R cis-eQTLs as instrumental variables to assess links with mood/behavior-related disorders. Summary-data MR (SMR) was then performed using GLP1R cis-eQTL data.

**Results:**

275,718 adverse events (AEs) associated with GLP-1 RAs were retrieved and analyzed. A mild signal for suicide-related AEs was observed only in the obesity indication subgroup (ROR:1.65, 95% CI: 1.28–2.12). Genetic evidence showed that GLP-1 RAs were likely associated with reduced risks of anxiety, depression, emotional lability, bipolar disorder, and suicide. Mediational analysis indicated that weight loss partially mediated the causal effects of GLP-1 RAs on depression and emotional lability, accounting for 18.28% (95% CI: 9.46–27.10%, *P* = 0.038) and 7.65% (95% CI: 5.66–9.64%, *P* < 0.001) of the total effects, respectively. SMR analysis showed that genetically predicted GLP1R expression was negatively associated with anxiety (OR: 0.79, 95% CI: 0.64–0.98, *P* = 0.031), with no significant associations for other emotional or behavioral outcomes.

**Conclusions:**

Both observational and MR analyses showed that patients treated with GLP-1 RAs may have no increased risk of emotional and behavioral disorders. Instead, genetic proxy activation of GLP-1 RAs may reduce the risk of anxiety, depression, and emotional lability.

## Introduction

The International Diabetes Federation reported that in 2021, there were 537 million people with diabetes globally, with the prevalence of diabetes estimated at 10.5% [1]. Over the past decade, with the continuous increase in the number of patients with diabetes and obesity worldwide, novel hypoglycemic drugs with innovative mechanisms of action have been introduced to the market, broadening the horizons for diabetes treatment. Glucagon-like peptide-1 receptor agonists (GLP-1 RAs) have seen a growing recommendation in the treatment of type 2 diabetes (T2D). This is attributed to their benefits in enhancing glycemic control and decreasing the risk of cardiovascular events [2, 3]. By transiently and glucose-dependently stimulating insulin release, suppressing glucagon secretion, and delaying gastric emptying, GLP-1 RAs effectively reduce blood glucose levels in individuals with diabetes [4, 5]. Due to their unique mechanism of lowering blood glucose, some GLP-1 RAs, such as liraglutide and exenatide, have now been approved by the FDA for obesity management.

With the widespread use of GLP-1 RAs, the reports of adverse drug reactions (ADRs) have gradually increased, which has drawn the attention of clinicians and drug regulatory authorities. The most frequently reported ADRs of GLP-1 RAs are gastrointestinal adverse drug reactions, including nausea, vomiting, and abdominal pain, which are closely related to the activation of both central and peripheral GLP-1 receptors [5, 6]. Worryingly, concerns have been raised regarding suicide and self-harm associated with the use of GLP-1 RAs. Reportedly, patients treated with GLP-1 RAs exhibited a 195% higher risk of major depression, a 108% increased risk for anxiety, and a 106% elevated risk for suicidal behavior [7]. Studies have also shown that while GLP-1 RAs lead to weight loss and blood glucose reduction, they may also induce emotional, physical, and psychological responses, which in turn could potentially impact suicidal ideation and self-harm [8, 9]. A study based on the World Health Organization (WHO) database also identified a signal associated with suicidal ideation for semaglutide [10].

However, a European cohort analyzed whether there was an association between GLP-1 RAs and an increased risk of suicide and self-harm. The study showed that there was no association between the use of GLP-1 RAs and an increased risk of suicide death, self-harm, depressive, and anxiety. Suicide deaths among users of GLP-1 RAs were rare [11]. Tsai et al. used claims data extracted from the National Health Insurance Research Database and found that diabetic patients receiving GLP-1 RAs treatment exhibited an even lower risk of anxiety in Taiwan [12]. Besides, preclinical studies (mainly in animal models) suggested that GLP-1 RAs may have potentially beneficial effects on mood and behavior [13]. A study has shown that GLP-1 RAs can slow the progression of Alzheimer’s disease (AD) by reducing β-amyloid protein deposition [14]. In McClean’s study, liraglutide reduced plaque formation in the brains of AD mouse models and preserved memory as well as synaptic plasticity [15].The activation of GLP-1 receptors induced the expression of β-endorphin in microglial cells, thereby exerting a neuroprotective effect in the central nervous system [16]. Studies have also reported that the effects of GLP-1 RAs are associated with complex interactions within the dopaminergic system. Activation of GLP-1 receptors can enhance the activity of dopaminergic neurons in the ventral tegmental area through a presynaptic mechanism [17]. Meanwhile, the expression of dopamine transporters on neurons in the limbic system and striatum increases, which leads to enhanced efficiency of dopamine reuptake and reduced availability of free dopamine in these regions. Given dopamine’s pivotal role in the brain’s reward system, such changes may result in an imbalance in dopaminergic neurotransmission [18]. It is hypothesized that this imbalance may lead to reward system dysfunction, which may potentially manifest as anhedonia and other psychiatric symptoms – a clinical observation that aligns with the psychiatric side effects noted in GLP-1 RAs therapy.

Currently, due to differences in the sources of analytical data and variations in the populations included in the studies, the existing research presents conflicting conclusions. Such discrepancies have led doctors to be concerned about how to choose the safest and most suitable drug treatment. Therefore, this study aims to explore the risk association between GLP-1 RAs and mood disorders based on the FDA Adverse Event Reporting System (FAERS), and subsequently determine causality through Mendelian randomization.

## Method

### Research design

We conducted a retrospective pharmacovigilance study using the data extracted from the FAERS database. The FAERS database is a large public database of adverse event (AE) reports submitted by healthcare professionals, pharmacists, and consumers worldwide. It is a recognized authoritative source for evaluating drug-related ADRs [19]. However, the limitations of this database may affect the interpretation of data on GLP-1 RAs. For example, since the reports are voluntary, it may lead to underreporting of some AEs, especially for ADRs, like mood swings that may not be easily noticed. In addition, there may be a bias in the dataset with a greater tendency to include reports of more severe adverse reactions, which may distort the prevalence of certain adverse reactions [20]. It should be noted that FAERS data cannot determine the causal relationship between drugs and ADRs; it can only indicate potential associations.

Mendelian randomization (MR) is a method that uses genetic tools to infer the potential causal relationship between exposure factors and outcomes, and the summary data-based Mendelian randomization (SMR) method is an innovative approach within the MR framework [21, 22]. Therefore, subsequent MR analysis to reveal the correlation between GLP-1 RAs represented by genetic factors and mood disorders is essential for confirming and expanding the preliminary findings based on the FAERS database.

### Data source

The study retrieved the recorded data from the FAERS database from the first quarter of 2004 to the fourth quarter of 2024 according to the procedures recommended by the FDA, and eliminated duplicate reports. This study employed both generic and brand names to comprehensively identify GLP-1 RAs, encompassing albiglutide, exenatide, liraglutide, dulaglutide, semaglutide, lixisenatide, and tirzepatide. These medications were subsequently designated as the primary suspected drugs (PS) associated with the reported AEs, thus ensuring a meticulous and inclusive approach to pinpointing potential causal agents in our analysis.

### Data processing

The Medical Dictionary for Regulatory Activities (MedDRA 27.1) was used to focus on the analysis of System Organ Classifications (SOC) in psychiatry-related ADRs to collect cases closely associated with the clinical manifestations of mood disorders [23]. This study identified and analyzed specific High-Level Group Terms (HLGTs) related to mood disorders, and classified psychiatric ADRs into several distinct categories – depression, hostility and aggression, suicide/self-harm, mental disorders, and non-infectious encephalopathy/delirium – by using Standardized MedDRA Queries (SMQs). Suicide can also be further categorized into suicidal ideation, suicide attempt, and completed suicide. Meanwhile, self-harm can be divided into self-harm ideation and self-harm behavior. To verify the internal consistency of the database, we used venlafaxine as a positive control drug for suicide or self-harm behaviors, given evidence of a significant association [24]. Similarly, according to the drug instructions and relevant research reports, the quinolone drug levofloxacin was selected as a positive control drug related to other mood disorders [25].

### Disproportionality analysis

Reporting odds ratio (ROR) compares the odds of reporting an event of interest in a specific drug to all other events relative to the reporting odds for other drugs in the FAERS database [26–28]. ROR method was also adopted to explore the potential association between GLP-1 RAs and mood disorders. The equations and criteria of the ROR algorithm were detailed in Supplementary Table S1. In the disproportionality analysis, a comprehensive assessment of the psychiatric AEs related to GLP-1 RAs was first conducted. Subsequently, using MedDRA, the study summarized four risk signals at the High-Level Term/High-Level Group Term (HLT/HLGT) level. This analytical approach ensured that the individual observations in the analysis were clinically relevant and could accurately represent the actual AEs of mood disorders, thereby enhancing the validity of our subsequent analysis. Taking into account the clinical applications of GLP-1 RAs, this study confined the indications to two categories: weight loss and blood glucose lowering, and conducted disproportionality analyses independently for each category. Additionally, given that epidemiological studies have demonstrated significant differences between males and females in the prevalence, clinical manifestations, and pathological mechanisms of mood disorders, such as the higher susceptibility of females to depression [29–31]. This study specifically established gender subgroup analyses to thoroughly investigate whether there are sex-specific distribution characteristics in the risk of mood disorders associated with GLP-1 receptor agonists. The study additionally incorporated non-GLP-1 RAs primarily indicated for weight loss or antidiabetic therapy into the analysis. Specifically, these drugs encompassed dipeptidyl peptidase-4 (DPP-4) inhibitors, sodium-glucose cotransporter 2 (SGLT2) inhibitors, metformin, sulfonylureas, orlistat, and phentermine. Since there was no direct evidence indicating an association between the above drugs and reports of mood disorders, they were regarded as negative controls [32].

### Selection of genetic instruments

Cis-expression quantitative trait loci (cis-eQTLs) of the drug target gene GLP1R were selected as a proxy for exposure to GLP-1 RAs [33, 34]. Specifically, the cis-eQTL summary level data of the GLP1R gene was obtained from the eQTLGen Consortium (https://eqtlgen.org/). Single-nucleotide polymorphisms (SNPs) significantly associated with GLP1R expression (*P* < 5.0 × 10^−8^) and with an effective allele frequency (EAF) exceeding 1% were selected as auxiliary variables [35]. In the next step, we performed clumping to reduce the impact of strong linkage disequilibrium, including only SNPs with weak linkage disequilibrium (r^2^ < 0.01). Additionally, the F values of the SNPs were calculated and SNPs with an F value less than 10 was excluded to avoid weak instrumental variable bias [36].

To determine the directionality of causality, we employed the MR-Steiger filtering method in the preliminary analysis to identify and exclude SNPs exhibiting reverse causality. This approach is based on the principle that a valid genetic instrument must explain more variance in the exposure trait than in the outcome phenotype. Genetic variants failing to meet this criterion were classified as exhibiting bidirectional pleiotropy and were excluded from subsequent analyses. Upon completing this quality control procedure, bidirectional two-sample MR analysis was reconducted using directionally validated instruments to ensure causal inference robustness.

### Outcome sources

Genome-wide association study (GWAS) datasets were retrieved from publicly accessible research sources. These datasets originated from approved studies conducted by diverse research institutions. To minimize potential research biases, GWAS data for exposures and outcomes were derived from distinct cohorts, with all populations in the datasets restricted to individuals of European ancestry. Summary statistics from FinnGen for type 2 diabetes (cases/controls: 82,878/403,489) and obesity (cases/controls: 31,499/468,693) were employed to assess the relationships between genetically instrumented GLP1R and glycemic control/weight reduction. In accordance with the findings of the FAERS pharmacovigilance analysis, outcomes associated with mood disorders were incorporated into the study. These outcomes include anxiety (cases/controls: 35,875/444,414), stress (cases/controls: 18,806/444,414), depression (cases/controls: 59,333/434,831), bipolar disorder (cases/controls: 8,946/434,831), emotionally unstable personality disorder (cases/controls: 5,464/486,464), and suicide (cases/controls: 11,538/488,810). All of these summary statistics were sourced from the FinnGen database. All of the above GWAS data were publicly available, see Supplementary Table S2 for details.

### Two-sample MR analysis

To elucidate the causal effect of GLP1R agonists on mood disorders, the study initially performed a two-sample MR analysis. In the two-sample MR analysis, the inverse variance weighting (IVW) method was the most effective method for detecting causal relationships [37]. The analysis results were primarily characterized by the odds ratio (OR) calculated via IVW method. Additionally, the study performed further sensitivity analyses using a series of approaches, including MR-Egger, MR pleiotropic residual analysis, and outlier-related analysis. The intercept test of MR–Egger regression was utilized in the study for the detection of pleiotropy. Specifically, a *p*-value <0.05 was considered indicative of horizontal pleiotropy [37]. The “TwoSampleMR” R package (version 0.6.14) was utilized to perform the aforementioned analyses [38].

### Mediation Mendelian randomization analysis

Although two-sample MR analysis initially explored the association between GLP1R and mood disorders, it remained unclear whether this association was related to the effects of GLP1R on improving blood glucose levels or reducing body weight. Therefore, we further conducted mediation analysis by combining the two-step MR method and the product-of-coefficients test. The two-step MR approach required causal relationships among exposure, mediator, and outcome; otherwise, the mediator might not exhibit true mediating effects [39]. The causal relationship between potential mediating factors and mood disorders would be further considered. If there was no causal relationship between potential mediating factors and outcomes, the causal effect from exposure factors to outcomes was unlikely to be mediated by this potential mediating factor. If a causal relationship was identified, we would apply the product-of-coefficients test to further examine the mediating effect [40].

### SMR analysis

Generally, when using cis-eQTLs as instrumental variables, it was necessary to adopt the summary data-based Mendelian randomization (SMR) method [41]. This is because relying solely on the IVW method for cis-eQTL analysis may yield false-positive results. Therefore, the study conducted a double analysis of the results of the “two-sample MR” through the SMR method. In the SMR analysis, the study selected SNPs significantly associated with GLP1R gene expression (*P* < 5 × 10^−8^) and filtered for those with a minor allele frequency > 0.01 to proxy for GLP-1 RAs exposure. Subsequently, the study used the SMR software (version 1.3.1) for allele coordination and analysis [42]. The ORs and 95% confidence intervals (CIs) were computed. To distinguish between pleiotropy and linkage, the heterogeneity in dependent instruments (HEIDI) test was used with a significance threshold set at *P* < 0.01.

## Results

### Descriptive analysis

**Figure 1.**
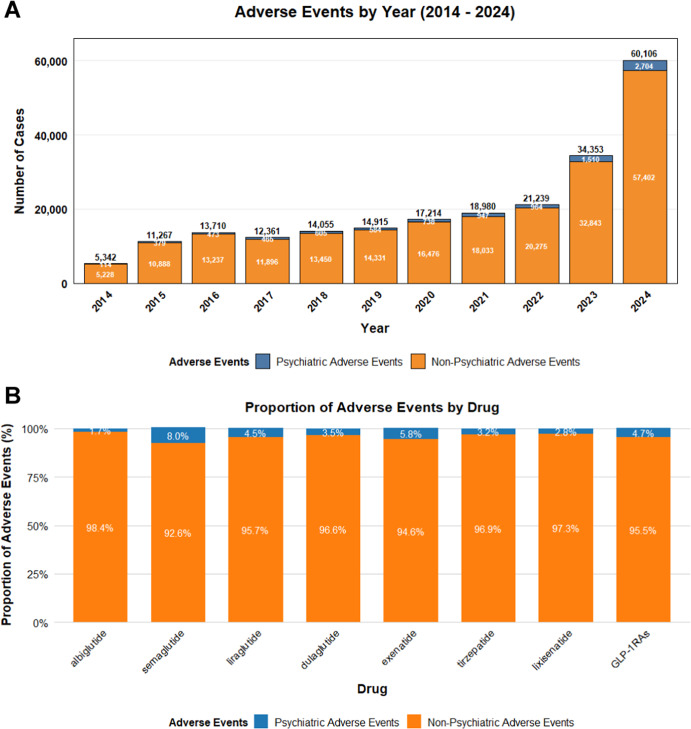
The statistical report on the number of adverse events related to GLP-1 receptor agonists from the FAERS database. (A) The number of reports related to GLP-1 receptor agonists in the FAERS database from Q1 2010 to Q4 2024. (B) The reports of mental-related adverse events and the proportion of other adverse events for GLP-1 receptor agonists in the FAERS database.

The study analyzed data from the FAERS database from Q1 2004 to Q4 2024. After removing duplicate entries, there were a total of 18,625,995 report data. A total of 275,718 AEs related to the target GLP-1 RAs were retrieved, among which 12,276 AEs were psychiatric-related adverse events. As shown in Figure 1, the time analysis indicated that the frequency of ADRs related to GLP-1 RAs had increased in recent years. The probability of psychiatric ADRs occurring with semaglutide and exenatide was higher than that with other drugs. Table 1 suggested the clinical characteristics of the population using GLP-1 RAs. Female patients (65.60%) had a higher probability of experiencing psychiatric ADRs than male patients (29.40%). 20.30% of patients had a body weight in the 50 – 100 kg range and 39.80% of patients were primarily aged between 18 and 64.9 years. The main sources of reports were consumers (80.00%) and physicians (8.50%). The United States constituted 87.30% of the report submissions, followed by the United Kingdom (3.80%) and Canada (1.10%) in descending order.

**Table 1.** Clinical characteristics of GLP-1 RAs drug-induced psychiatric adverse events reported in FAERS database

Characteristic	GLP-1 RAs	albiglutide	semaglutide	liraglutide	dulaglutide	exenatide	tirzepatide	lixisenatide
	(N = 12276)	(N = 140)	(N = 2726)	(N = 1232)	(N = 2283)	(N = 4097)	(N = 1725)	(N = 73)
**Gender**								
Female	8059 (65.6%)	90 (64.3%)	1750 (64.2%)	841 (68.3%)	1388 (60.8%)	2783 (67.9%)	1163 (67.4%)	44 (60.3%)
Male	3604 (29.4%)	47 (33.6%)	807 (29.6%)	364 (29.5%)	747 (32.7%)	1242 (30.3%)	375 (21.7%)	22 (30.1%)
Missing	613 (5.0%)	3 (2.1%)	169 (6.2%)	27 (2.2%)	148 (6.5%)	72 (1.8%)	187 (10.8%)	7 (9.6%)
**Weight**								
<50 kg	21 (0.2%)	0 (0%)	1 (0.0%)	2 (0.2%)	2 (0.1%)	11 (0.3%)	4 (0.2%)	1 (1.4%)
>100 kg	1537 (12.5%)	4 (2.9%)	257 (9.4%)	130 (10.6%)	66 (2.9%)	958 (23.4%)	121 (7.0%)	1 (1.4%)
50 ~ 100 kg	2494 (20.3%)	7 (5.0%)	451 (16.5%)	166 (13.5%)	91 (4.0%)	1586 (38.7%)	189 (11.0%)	4 (5.5%)
Missing	8224 (67.0%)	129 (92.1%)	2017 (74.0%)	934 (75.8%)	2124 (93.0%)	1542 (37.6%)	1411 (81.8%)	67 (91.8%)
**Age**								
<18 years	25 (0.2%)	0 (0%)	9 (0.3%)	10 (0.8%)	4 (0.2%)	1 (0.0%)	1 (0.1%)	0 (0%)
>85 years	50 (0.4%)	0 (0%)	19 (0.7%)	1 (0.1%)	18 (0.8%)	8 (0.2%)	3 (0.2%)	1 (1.4%)
18 ~ 64.9 years	4880 (39.8%)	52 (37.1%)	1123 (41.2%)	555 (45.0%)	568 (24.9%)	1685 (41.1%)	876 (50.8%)	21 (28.8%)
65 ~ 85 years	2490 (20.3%)	24 (17.1%)	541 (19.8%)	255 (20.7%)	432 (18.9%)	976 (23.8%)	241 (14.0%)	21 (28.8%)
Missing	4831 (39.4%)	64 (45.7%)	1034 (37.9%)	411 (33.4%)	1261 (55.2%)	1427 (34.8%)	604 (35.0%)	30 (41.1%)
**Reporter’s type**								
Consumer	9816 (80.0%)	129 (92.1%)	1962 (72.0%)	866 (70.3%)	2031 (89.0%)	3196 (78.0%)	1576 (91.4%)	56 (76.7%)
Physician	1040 (8.5%)	8 (5.7%)	366 (13.4%)	203 (16.5%)	81 (3.5%)	327 (8.0%)	49 (2.8%)	6 (8.2%)
Other health-professional	191 (1.6%)	0 (0%)	13 (0.5%)	65 (5.3%)	27 (1.2%)	84 (2.1%)	0 (0%)	2 (2.7%)
Pharmacist	269 (2.2%)	1 (0.7%)	118 (4.3%)	38 (3.1%)	49 (2.1%)	40 (1.0%)	23 (1.3%)	0 (0%)
Lawyer	61 (0.5%)	0 (0%)	1 (0.0%)	3 (0.2%)	28 (1.2%)	5 (0.1%)	23 (1.3%)	1 (1.4%)
Missing	486 (4.0%)	2 (1.4%)	17 (0.6%)	20 (1.6%)	9 (0.4%)	432 (10.5%)	6 (0.3%)	0 (0%)
**Serious outcome**								
Hospitalization-initial or prolonged	1330 (10.8%)	8 (5.7%)	282 (10.3%)	168 (13.6%)	237 (10.4%)	484 (11.8%)	144 (8.3%)	7 (9.6%)
Disability	186 (1.5%)	1 (0.7%)	89 (3.3%)	20 (1.6%)	20 (0.9%)	24 (0.6%)	30 (1.7%)	2 (2.7%)
Life-threatening	248 (2.0%)	1 (0.7%)	107 (3.9%)	27 (2.2%)	24 (1.1%)	54 (1.3%)	35 (2.0%)	0 (0%)
Death	124 (1.0%)	0 (0%)	36 (1.3%)	15 (1.2%)	17 (0.7%)	47 (1.1%)	8 (0.5%)	1 (1.4%)
Required intervention to prevent permanent impairment/damage	64 (0.5%)	0 (0%)	35 (1.3%)	3 (0.2%)	2 (0.1%)	8 (0.2%)	16 (0.9%)	0 (0%)
Congenital anomaly	0 (0%)	0 (0%)	0 (0%)	0 (0%)	0 (0%)	0 (0%)	0 (0%)	0 (0%)
Other serious	2049 (16.7%)	14 (10.0%)	709 (26.0%)	265 (21.5%)	336 (14.7%)	349 (8.5%)	355 (20.6%)	21 (28.8%)
**Reported countries (top five)**								
America	10720 (87.3%)	138 (98.6%)	2158 (79.2%)	943 (76.5%)	2070 (90.7%)	3910 (95.4%)	1444 (83.7%)	57 (78.1%)
Britain	462 (3.8%)	0 (0%)	130 (4.8%)	40 (3.2%)	25 (1.1%)	28 (0.7%)	239 (13.9%)	0 (0%)
Canada	138 (1.1%)	0 (0%)	95 (3.5%)	36 (2.9%)	1 (0.0%)	4 (0.1%)	1 (0.1%)	1 (1.4%)
Australia	97 (0.8%)	0 (0%)	48 (1.8%)	14 (1.1%)	15 (0.7%)	16 (0.4%)	4 (0.2%)	0 (0%)
Brazil	90 (0.7%)	0 (0%)	28 (1.0%)	40 (3.2%)	9 (0.4%)	6 (0.1%)	1 (0.1%)	6 (8.2%)

### ADRs signal distribution based on SOC

**Figure 2.**
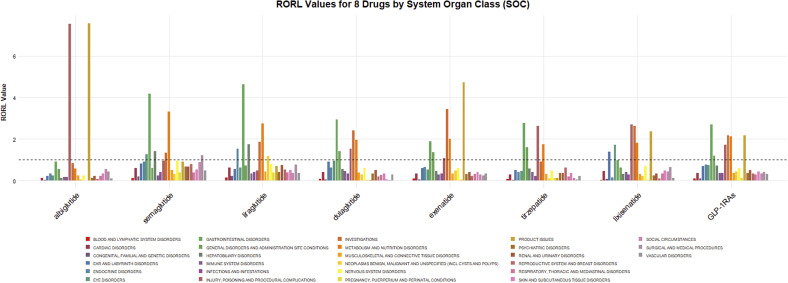
RORL (95%CI lower limit) of adverse reactions related to GLP-1 receptor agonists at SOC level in FAERS database.

275,718 cases of adverse drug events (ADEs) related to GLP-1 RAs were classified according to the SOC. Figure 2 visualized the signals for the 27 SOC categories. The ADR signals of GLP-1 RAs mainly concentrated on six SOCs: gastrointestinal system diseases (OR:2.70, 95% CI: 2.69–2.72), abnormal clinical examinations (OR:2.19, 95% CI:2.18–2.21), metabolic disorders (OR: 2.15, 95% CI: 2.12–2.17), product issues (OR: 2.20, 95% CI: 2.17–2.23), iatrogenic injuries (OR:1.73, 95% CI: 1.72–1.75), and systemic reactions (OR: 1.21, 95% CI:1.20–1.22). However, the psychiatric disorders category of SOC did not show a reliable signal (OR: 0.37, 95% CI: 0.37–0.38).

### ADRs signal detection based on PT related to psychiatric disorders

**Figure 3.**
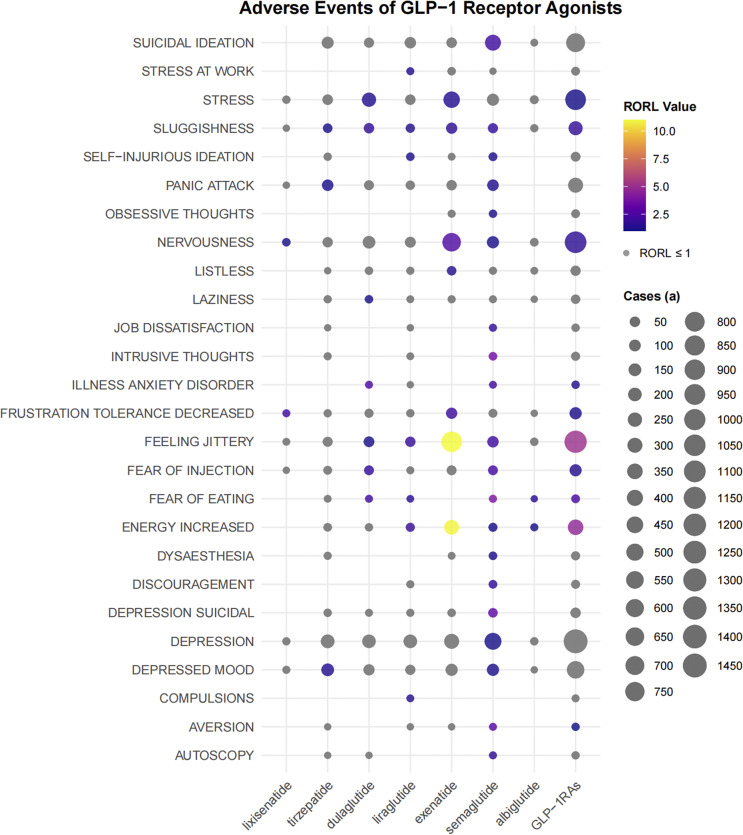
The bubble chart shows the signal values of emotional disorders related adverse events of different GLP-1 receptor agonists in FAERS database. Colored bubbles indicate that the RORL value of the preferred term is greater than 1, and gray bubbles indicate that the RORL value is less than or equal to 1; The bubble size represents the number of cases of emotional disorder-related adverse events, and the larger the bubble, the more cases of the adverse events.

We analyzed each preferred term (PT) related to psychiatric disorders. As shown in Figure 3, GLP-1 RAs exhibited strong ADR signals in terms of stress, sluggishness, nervousness, illness anxiety disorder, decreased frustration tolerance, feeling jittery, fear of injection, fear of eating, increased energy, and aversion. There were differences in the disproportional signals between some drugs. For example, semaglutide had detected a signal for suicidal ideation, while no such PT signal had been detected in other drugs.

### ADRs signal analysis based on HLGT and SMQs

**Figure 4.**
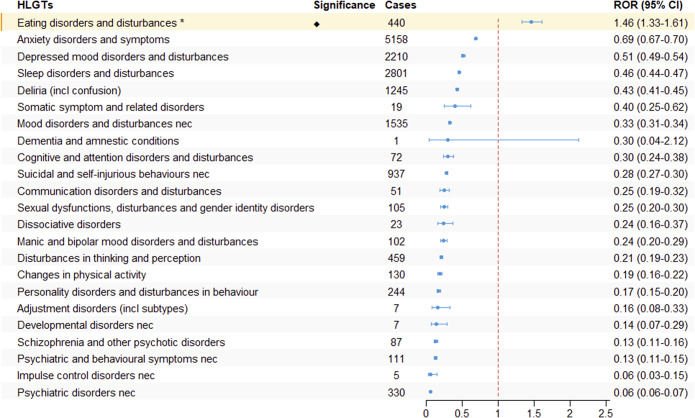
The signal strength of ADRs of GLP-1 receptor agonists at the HLGT level in FAERS database.

We further systematically classified the PTs under the SOC of psychiatric disorders using HLGTs. As shown in Figure 4, only “Eating Disorders and Disturbances” in the HLGTs terminology set showed adverse reaction signals (ROR: 1.46, 95% CI: 1.33–1.61).

**Figure 5.**
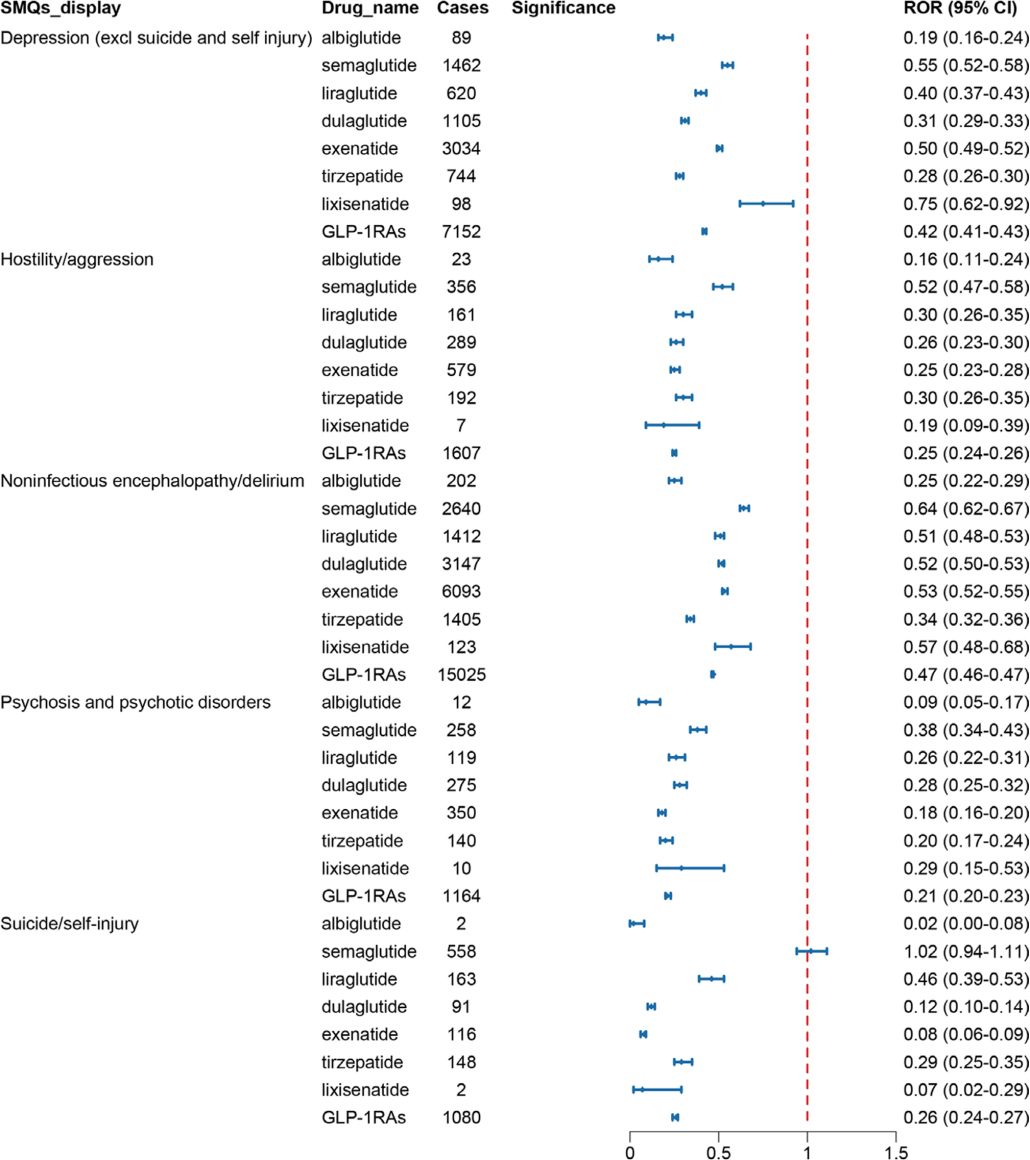
The signal strength of ADRs of GLP-1 receptor agonists at the SMQ level in FAERS database.

Standardized MedDRA Queries (SMQs) utilized carefully chosen MedDRA terminology to focus on specific medical conditions, which included PTs related to signs, symptoms, diagnoses, and other relevant clinical manifestations. The scope of SMQs could be broad or narrow. First, a broad scope was selected to screen all psychiatric-related ADRs with positive signals. These reactions were ultimately classified into five SMQs: depression (excluding suicide and self-harm), suicide/self-harm, hostility/aggression, psychosis and psychiatric disorders, and non-infectious encephalopathy/delirium. Figure 5 presented their reporting frequencies alongside ROR analysis, and no significant disproportional signals were identified.

### ADRs signal analysis based on indications of GLP-1 RAs

We conducted a pooled analysis of these AEs based on HLGT/HLT levels and narrower SMQs scopes and performed disproportionality analyses for each ADR group to enhance specificity. The positive control drugs venlafaxine (mood disorders: ROR:3.39, 95% CI: 3.29–3.48; suicide: ROR:7.32, 95% CI:7.13–7.52) and levofloxacin (anxiety: ROR:1.56, 95% CI: 1.48–1.63; depression: ROR:1.51, 95% CI: 1.42–1.61) confirmed the internal validity of the FAERS database and the specificity of each adverse reaction group.

**Figure 6.**
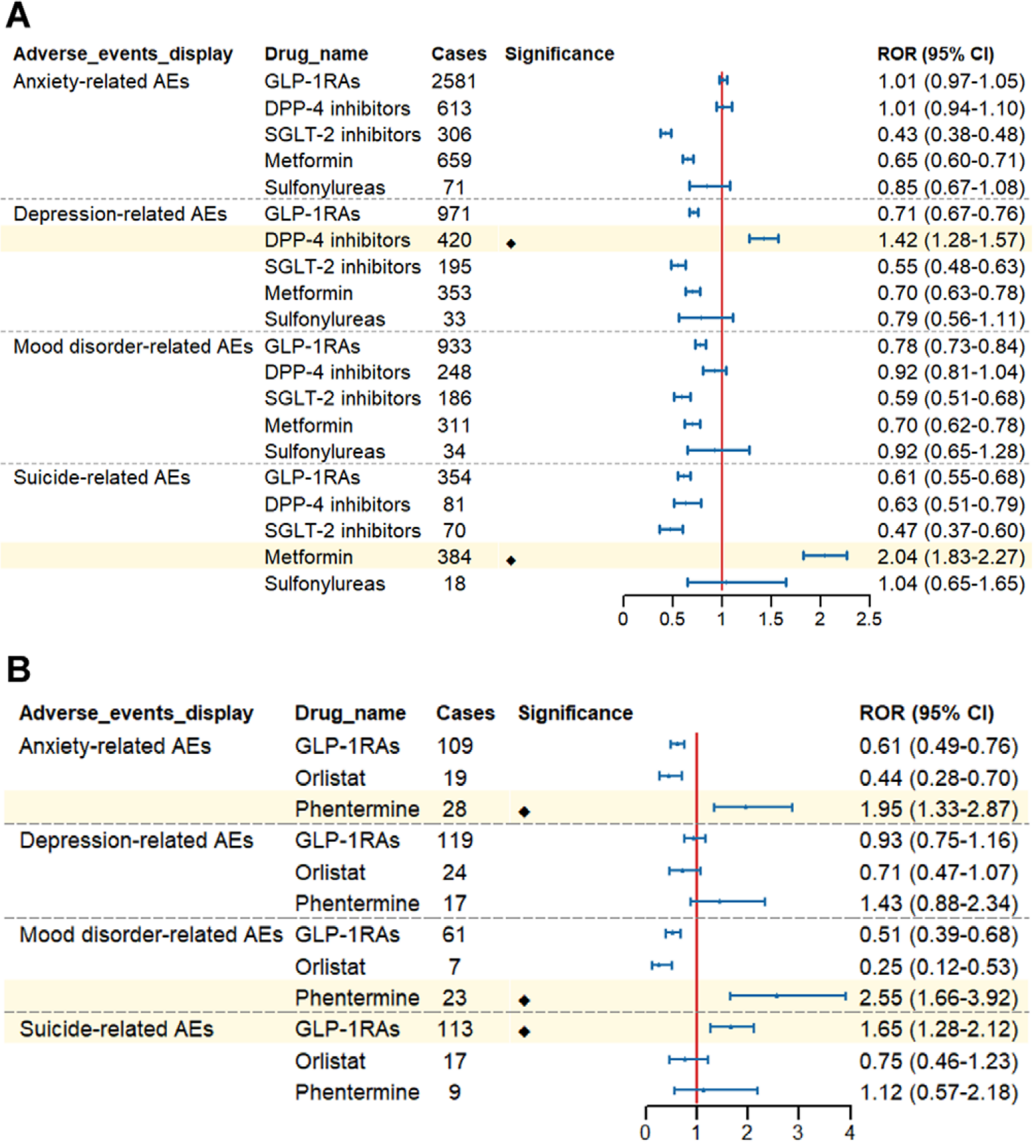
Forest plot of risk signals for adverse reactions related to four groups of specific emotional disorders. (A) The ROR of different glucose-lowering-drug-related emotional disorders AEs. (B) The ROR of different weight-loss-drug-related emotional disorders AEs.

Based on the indications of GLP-1 RAs, we performed subgroup analyses. In the subgroup analysis focusing on drug classes for diabetes treatment, DPP-4 inhibitors showed signals associated with depression (ROR:1.42, 95% CI: 1.28–1.57), metformin showed signals associated with suicide (ROR:2.04, 95% CI:1.83–2.27), while other antidiabetic drugs, including GLP-1 RAs, did not show relevant signal risks (Figure 6A). When diabetic patients were further divided into male and female subgroups, no ADR signals were similarly observed in terms of anxiety, depression, mood disorders, suicide, and self-harm in either male or female subgroups. (Supplementary Table S3).

Interestingly, in the subgroup analysis of drug classes for treating obese patients, GLP-1 RAs were observed to show signals associated with suicide (ROR:1.65, 95% CI:1.28–2.12), with no significant signals detected for anxiety, depression, mood disorders, and self-harm. In contrast, phentermine demonstrated signals associated with anxiety (ROR: 1.95, 95% CI: 1.33–2.87) and mood disorders (ROR: 2.55, 95% CI: 1.66–3.92). Additionally, no signals for the above four specific ADR groups were detected in other weight-loss medications (Figure 6B). Further analysis of the suicide signals associated with GLP-1 RAs revealed that GLP-1 RAs primarily exhibited signals in suicidal ideation (ROR:2.09, 95% CI:1.55–2.82). Additionally, we further analyzed gender differences in the signals of suicidal ideation and found that both male obese patients (ROR:2.15, 95% CI:1.23–3.75) and female obese patients (ROR:2.20, 95% CI:1.52–3.18) showed signals in suicidal ideation (Supplementary Table S4).

### Two-sample MR analysis of GLP-1 RAs and mood disorders

**Figure 7.**
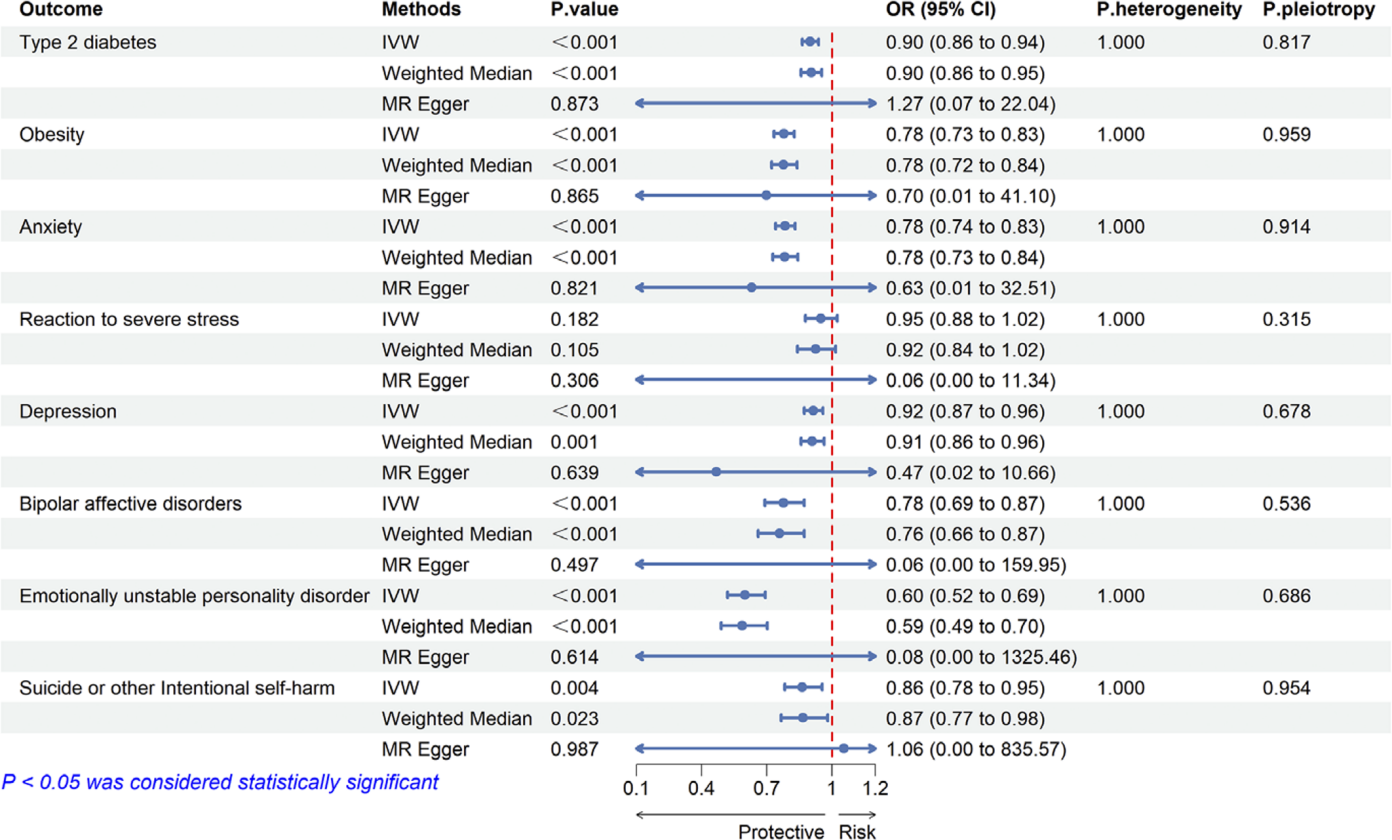
Two-sample Mendelian randomized analysis of GLP1R and T2D, BMI and emotional disorders.

We selected 22 significant cis-eQTL SNPs from eQTLGen as instrumental variables (IVs) for the drug target GLP1R gene, with F-statistics all exceeding 47, indicating no weak instrument-induced bias in our study (Supplementary Table S5). MR analysis showed that GLP-1 RAs may be associated with a reduced risk of T2D (OR:0.900, 95% CI: 0.863–0.939, *P* < 0.001) and obesity (OR:0.779, 95% CI:0.733–0.827, *P* < 0.001) (Figure 7). There was no evidence of heterogeneity or horizontal pleiotropy (Supplementary Tables S6, S7), suggesting that the selected IVs were valid.

The results of two-sample MR analysis showed that GLP-1 RAs reduced the risk of mood disorder-related ADRs. The results based on the IVW method were as follows: anxiety (OR: 0.784, 95% CI: 0.740–0.831, *P* < 0.001), depression (OR: 0.915, 95% CI: 0.873–0.958, *P* < 0.001), bipolar disorder (OR: 0.777, 95% CI: 0.692–0.873, *P* < 0.001), emotionally unstable personality disorder (OR: 0.600, 95% CI: 0.519–0.693, *P* < 0.001), and suicide (OR: 0.864, 95% CI: 0.783–0.954, *P* = 0.003) (Figure 7). We assessed the heterogeneity of effect sizes for selected genetic IVs using Cochran’s Q test and further evaluated horizontal pleiotropy using the intercept from MR-Egger regression. The MR-PRESSO analysis was executed to detect outliers and to adjust for potential heterogeneity and horizontal pleiotropy. The sensitivity analysis yielded robust results: No significant heterogeneity or horizontal pleiotropy was detected (Cochran’s Q *P* > 0.05 and MR-Egger intercept *P* > 0.05) (Supplementary Tables S6, S7). MR-Steiger directionality testing further confirmed that the direction of causality aligned with our hypothesis (all *P* < 0.05), effectively ruling out reverse causation (Supplementary Table S8). Leave-one-out analysis demonstrated that the observed associations were not driven by any single SNP.

### Mediation Mendelian randomization analysis

**Figure 8.**
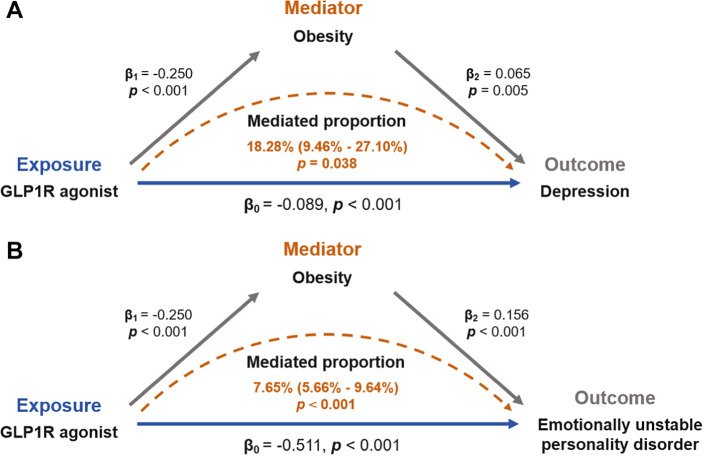
Obesity mediates the risk association between GLP-1 receptor agonists and emotional disorders.

Mediation MR analysis was employed to elucidate the mechanistic pathways underlying the causal association between GLP-1 RAs and mood disorders. Specifically, we investigated the potential mediating roles of T2D and obesity in this relationship. We found no significant association between T2D and mood disorders (Supplementary Table S6). Specifically, the effect of GLP-1 RAs in reducing the risk of mood disorders may not be related to their hypoglycemic mechanism. However, there may be a causal relationship between obesity and depression (OR: 1.067, 95% CI: 1.019–1.117, *P* = 0.005) and emotional instability (OR: 1.169, 95% CI: 1.076–1.271, *P* < 0.001). Detailed results were tabulated in Supplementary Table S7. Finally, we reported causal effects indicating that the role of GLP-1 RAs in reducing the risk of depression and emotional instability may be mediated by BMI reduction, as shown in Figure 8. The coefficient product test using the delta method further supported the mediating effects of obesity.

### Summary Mendelian randomization (SMR) analysis of GLP1R and mood disorders

**Figure 9.**
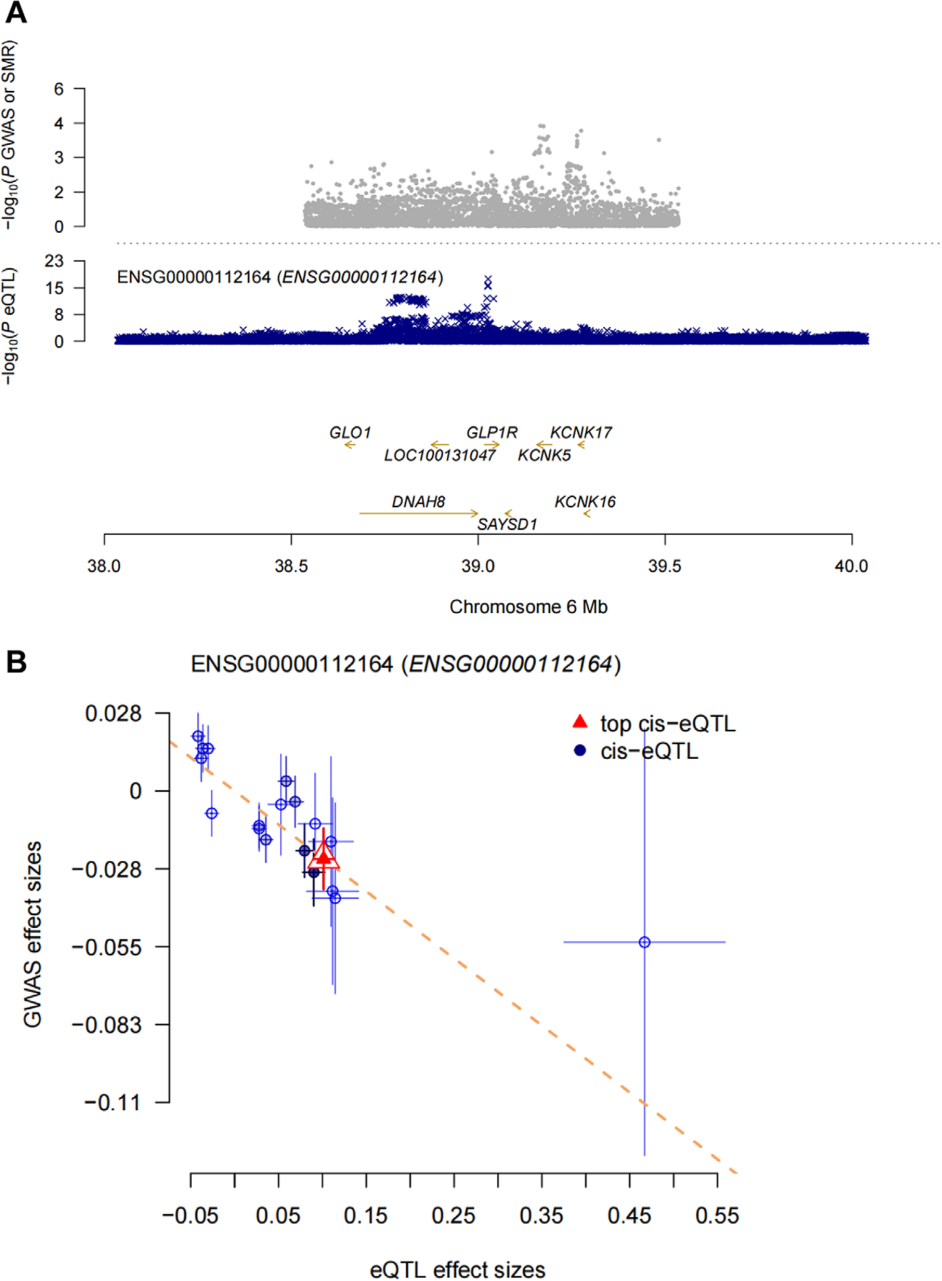
SMR analysis. (A) SMR locus plot. (B) SMR effect plot.

When cis-eQTLs were used as IVs, there may be an inclusion of too many inappropriate IVs, in which case the IVW weighting method may lead to false-positive results. Therefore, we performed SMR analysis to provide more reliable insights into the potential causal relationship between GLP-1 RAs and mood disorder-related ADRs. The SMR analysis showed that each standard deviation increase in GLP1R gene expression (the target of the GLP-1 receptor, probe: ENSG00000112164) was associated with a reduced risk of anxiety (OR:0.79, 95% CI:0.64–0.98, *P* = 0.031) (Figure 9). However, no significant associations were observed between GLP1R gene expression and other mood disorder-related ADRs based on the current data (Supplementary Table S9). The HEIDI test indicated that the association between GLP1R and anxiety was not driven by linkage disequilibrium (Supplementary Table S9).

## Discussion

This study provided a novel perspective on exploring the association between GLP-1 RAs and mood disorders using publicly accessible databases. First, we conducted data mining on AEs of mood disorders caused by GLP-1 RAs from the FAERS database. Briefly, our findings did not detect a high signal for AEs of mood disorders associated with GLP-1 RAs. We then performed two-sample MR and SMR analyses to estimate the association between gene expression of GLP-1 RAs’ drug target and mood disorders using eQTL data and GWAS summary statistics. The two-sample MR analysis showed that GLP-1 RAs may reduce the risk of mood disorder-related ADRs (Figure 7). Mediation MR analysis suggests that GLP1 RAs may reduce depression and emotionally unstable personality disorder through the mediator of obesity (Figure 8). Additionally, the SMR analysis revealed that each one-standard-deviation increase in GLP1R gene expression was associated with a reduced risk of anxiety, while no significant associations were found between GLP1R gene expression and other mood disorder-related ADRs. Our analysis suggested that GLP-1 RAs may not be associated with an increased risk of mood disorder-related AEs. Overall, our results provided real world evidence but failed to provide strong genetic evidence for the association between GLP-1 RAs and mood disorders.

As two widely used GLP-1 RAs, semaglutide and liraglutide have attracted increasing attention. Both agents can enhance glucose-dependent insulin secretion, reduce glucagon secretion, and delay gastric emptying [43–45]. Owing to their unique hypoglycemic mechanism, these drugs – initially developed for diabetes treatment – have been extensively used in patients with obesity and have obtained approval from the FDA. Compared with traditional insulin, semaglutide and liraglutide (as novel hypoglycemic agents) exhibit distinct advantages. Studies have reported that semaglutide and liraglutide are less likely to cause hypoglycemia and are more convenient to use [10, 45]. A clinical trial investigating the hypoglycemic efficacy of semaglutide versus liraglutide showed that semaglutide was superior to liraglutide in reducing glycated hemoglobin (HbA1c) levels and body weight [46]. Another randomized controlled clinical trial specifically focusing on their weight loss effects demonstrated that, in adults with overweight or obesity without diabetes, once-weekly subcutaneous injection of semaglutide resulted in significantly greater weight loss at 68 weeks compared with once-daily subcutaneous injection of liraglutide, and the incidence of gastrointestinal adverse reactions was similar between the two groups [47]. Although both clinical trials monitored the occurrence of gastrointestinal adverse reactions, they barely mentioned adverse reactions related to mood, suicide, or self-harm.

Numerous studies have suggested that GLP-1 RAs may lead to mood-related adverse reactions. For example, Edy Kornelius et al. conducted a large community-based cohort study to investigate the impact of GLP-1 RAs on the risk of mental disorders, such as depression, anxiety, and suicidal behavior in obese patients. The study found a significant 98% increased risk of any mental disorder associated with GLP-1 RA treatment. Notably, patients using GLP-1 RAs had a 195% higher risk of major depression, a 108% higher risk of anxiety, and a 106% higher risk of suicidal behavior [7]. Georgios Schoretsanitis et al. performed disproportionality analysis on the WHO global database of suspected ADRs and identified a signal for semaglutide-related suicidal ideation, while no such signal was observed for liraglutide [10]. Growing evidence has suggested that GLP-1 RAs may affect patients’ mood, leading to anxiety, depression, and even suicidal tendencies. However, some studies have reported contradictory results. A FAERS database analysis of suicidal tendencies associated with GLP-1 RAs showed disproportionate reports of suicidal ideation and “depression/suicide” for semaglutide and liraglutide, but no disproportionate signals for suicidal behavior, attempted suicide, or suicide death across all FDA-approved GLP-1 RAs [48]. Importantly, a recent retrospective study had found that GLP-1 RAs reduced suicidal ideation in large cohorts of overweight or T2D patients compared to non-GLP-1R agonist anti-obesity or anti-diabetes medications [49]. Furthermore, a population-based longitudinal study found that patients with T2D who received GLP-1 RA therapy had a reduced risk of depression and anxiety [12]. A meta-analysis also showed that GLP-1 RAs could reduce depression severity scores, with a greater impact on diabetic participants [50]. By expanding the scope of adverse reactions to include “depression (excluding suicide and self-harm), suicide/self-injury, hostility/aggression, psychosis and mental disorders, and non-infectious encephalopathy/delirium” as mood disorders, and conducting subgroup analyses by GLP-1 RAs indications, our study showed no significant risk signals for GLP-1 RAs. Notably, in the subgroup analysis for obesity indications, GLP-1 RAs exhibited a suicide ideation-related signal (ROR: 2.09, 95% CI: 1.55–2.82). There was no gender difference in this risk signal. Specifically, the risk signal related to suicidal ideation was detected in both male and female patients with obesity.

It is well-established that adverse reaction analyses based on the FAERS database have the following limitations: passive reporting systems, heterogeneous data quality, difficulty in eliminating confounding factors, and an inability to establish causality [51, 52]. Current studies suggesting that GLP-1 RAs may cause mood problems only indicated a potential association between GLP-1 RAs and mood disorders, lacking sufficient evidence to prove causality. Additionally, some study results also suggest that GLP-1 RAs reduce the occurrence of anxiety, depression, suicidal behaviors, and so on [12, 49, 50]. Animal studies have also shown that GLP-1 RAs alleviated neurological symptoms. Semaglutide has been proven to effectively relieve depression and anxiety, while significantly improving cognitive function. Semaglutide treatment could protect synaptic plasticity, reverse hippocampal neuroinflammation induced by high-fat diet, promote the activation of insulin signaling pathways, and exert neuroprotective effects. It could also reduce astrogliosis and microgliosis in the dentate gyrus region of the hippocampus, prevent the impairment of pro-opiomelanocortin and G-protein-coupled receptor 43 expression caused by T2D, and simultaneously increase the number of NeuN+ and GLP-1R neurons in the hippocampus [53]. Another study indicated liraglutide treatment protected synaptic plasticity and reversed the inhibition of hippocampal long-term potentiation induced by corticosterone (CORT) administration, demonstrating its synaptic protective effect. The study also found that liraglutide treatment increased the cell density of immature neurons in the subgranular dentate gyrus region of the hippocampus. Liraglutide prevented CORT-induced damage while increasing the level of phosphorylated glycogen synthase kinase 3β (p-GSK3β) in the hippocampus [54]. Clinical studies and animal trials have shown that GLP-1 RAs may be associated with neuroprotection, thereby alleviating mood disorders. To further investigate the causal relationship between GLP-1 RAs and mood disorders, we employed MR and SMR analyses using eQTL data and GWAS summary statistics. The MR analysis demonstrated that GLP-1 RAs may reduce the risk of mood disorder-related ADRs, including anxiety, depression, bipolar disorder, emotionally unstable personality disorder, and suicide. The SMR analysis showed that increased GLP1R gene expression was associated with a reduced risk of anxiety, but no significant associations were observed with other mood disorder-related adverse reactions. These findings corroborated our FAERS-based results, indicating that GLP-1 RAs may not increase the risk of mood disorders.

Why do some other studies based on the FAERS database yield positive results indicating an association between GLP-1 receptor agonists (GLP-1 RAs) and mood-related adverse reactions? As previously mentioned, the FAERS database’s inherent limitation in eliminating confounding factors may explain why positive associations are often reported. In reality, GLP-1 RAs are primarily used to treat diabetes and obesity. However, these two patient populations may inherently have pre-existing emotional issues. A German study showed that the prevalence of affective disorders in diabetic patients was twice as high as in non-diabetic populations [55]. Diabetes has been established as a contributing factor to various psychological disorders in patients, including a distinct condition termed diabetes distress (DD) [56]. Characterized by emotional disturbances, stress, guilt, and treatment avoidance, DD affected approximately 45% of patients worldwide-a prevalence underscoring its clinical significance [57]. Obesity, similar to diabetes, is also associated with emotional issues in patients.

A Swedish national study has shown that girls in the obese cohort have a 43% higher risk of anxiety and depression compared to girls in the general population (adjusted HR: 1.43, 95% CI: 1.31–1.57; *P* < 0.0001). A similar risk was observed in boys (adjusted HR: 1.33, 95% CI: 1.20–1.48; *P* < 0.0001) [58]. Additionally, patients with conditions such as diabetes or obesity often experience fear of disease recurrence or symptom exacerbation [59]. Therefore, the associations between GLP-1 RAs and mood disorders (such as anxiety and depression) identified in FAERS analyses may likely stem from the underlying diseases rather than the drugs themselves. Our causal analyses suggested that GLP-1 RAs could alleviate certain mood disorders, which might be attributed to either the improvement of patients’ conditions following GLP-1 RA treatment, or the neuroprotective effects of GLP-1 RAs themselves on neurons. Additionally, the results of mediation analysis suggested that the alleviation of mood disorders by GLP-1 RAs may be mediated by obesity as an intermediate factor.

When interpreting our results cautiously, we must also take limitations into account, which are consistent with those of other MR methods that use aggregated data. First, all data involved in the analysis are derived from individuals of European ancestry, which limits the generalizability of the MR study results to other ethnic groups. Second, in this study, cis-eQTLs of the *GLP1R* gene were used as proxy variables for GLP-1 RAs exposure, and there may be certain differences between this approach and the agonistic effects of actual pharmaceutical treatments. Finally, precisely because the IVs for GLP-1 RAs were derived from the eQTLGen study, these IVs were inherently limited to specific chromosomal regions. In contrast, the variations in obesity and T2D were genome-wide. This limitation restricted the availability of valid IVs for multivariable MR [60, 61]. Therefore, we prioritized conducting two-step mediational MR and SMR analyses, which confirmed that GLP-1 RAs may not induce mood disorders.

In conclusion, our real-world study demonstrates that the use of GLP-1 RAs may not be associated with an increased risk of mood/behavior disorders, including depression, anxiety, and suicidal behavior. Additionally, GLP-1 RAs may alleviate symptoms of related adverse reactions. These findings may enhance clinicians’ confidence in utilizing this class of drugs for the treatment of diabetes and obesity. Nevertheless, future studies should further collect real-world data outcomes or conduct rigorous clinical trials to thoroughly investigate the relationship between GLP-1 RAs and mood disorders.

## Supporting information

10.1192/j.eurpsy.2025.10125.sm001 Zheng et al. supplementary material Zheng et al. supplementary material

## Data Availability

The data supporting the findings of this study were derived from the following publicly available resources: the U.S. FDA Adverse Event Reporting System (FAERS) (https://fis.fda.gov/extensions/FPD-QDE-FAERS/FPD-QDE-FAERS.html) and the FinnGen database (https://www.finngen.fi/en/access_results). All generated and/or analyzed datasets during this study are also included within this article and its supplementary information. The custom code used is available from the corresponding author upon reasonable request.

## Supplementary material

The supplementary material for this article can be found at http://doi.org/10.1192/j.eurpsy.2025.10125.
