# Supplementary material for: Investigating the association between GLP-1 receptor agonists and mood disorders: A study integrating real-world data and Mendelian randomization
Source: Eur Psychiatry. 2025 Dec 3;69(1):e6. doi: 10.1192/j.eurpsy.2025.10125 (PMC12816927; doi:10.1192/j.eurpsy.2025.10125)
Supplement: Zheng et al. supplementary material [file S0924933825101259sup001.docx]

| **Table S1. The Reporting Odds Ratio (ROR) is the main algorithm used for signal detection.** | | |
| --- | --- | --- |
| **Algorithms** | **Equation** | **Criteria** |
| ROR | ROR=ad/b/c | lower limit of 95% CI>1, N≥3 |
|  | 95%CI=e^ln(ROR)±1.96(1/a+1/b+1/c+1/d)^0.5^ |  |
| Equation: a, number of reports containing both the target drug and target adverse drug reaction; b, number of reports containing other adverse drug reaction of the target drug; c, number of reports containing the target adverse drug reaction of other drugs; d, number of reports containing other drugs and other adverse drug reactions. 95%CI, 95% confidence interval; N, the number of reports. | | |

| **Table S2. Detailed information for genome-wide association studies involved in the present Mendelian Randomization study.** | | | | | |
| --- | --- | --- | --- | --- | --- |
| **Phenotype** | **Data source** | **num_cases** | **num_controls** | **Ancestry** | **Path_https** |
| Type 2 diabetes | Finngen_R12 | 82878 | 403489 | European | https://storage.googleapis.com/finngen-public-data-r12/summary_stats/release/finngen_R12_T2D.gz |
| Obesity | Finngen_R12 | 31499 | 468693 | European | https://storage.googleapis.com/finngen-public-data-r12/summary_stats/release/finngen_R12_E4_OBESITY.gz |
| All anxiety disorders | Finngen_R12 | 35875 | 444414 | European | https://storage.googleapis.com/finngen-public-data-r12/summary_stats/release/finngen_R12_F5_ALLANXIOUS.gz |
| Other reaction to severe stress, and adjustment disorders | Finngen_R12 | 18806 | 444414 | European | https://storage.googleapis.com/finngen-public-data-r12/summary_stats/release/finngen_R12_F5_STRESSOTH.gz |
| Depression | Finngen_R12 | 59333 | 434831 | European | https://storage.googleapis.com/finngen-public-data-r12/summary_stats/release/finngen_R12_F5_DEPRESSIO.gz |
| Bipolar affective disorders | Finngen_R12 | 8946 | 434831 | European | https://storage.googleapis.com/finngen-public-data-r12/summary_stats/release/finngen_R12_F5_BIPO.gz |
| Emotionally unstable personality disorder | Finngen_R12 | 5464 | 486464 | European | https://storage.googleapis.com/finngen-public-data-r12/summary_stats/release/finngen_R12_F5_EMOPER.gz |
| Suicide or other Intentional self-harm | Finngen_R12 | 11538 | 488810 | European | https://storage.googleapis.com/finngen-public-data-r12/summary_stats/release/finngen_R12_VWXY20_SUICI_OTHER_INTENTI_SELF_H.gz |

| **Table S3.** **Gender subgroup analysis of risk signals for adverse reactions related to four specific mood disorders.** | | | | | | | |
| --- | --- | --- | --- | --- | --- | --- | --- |
| **Drug indications** | **Gender** | **Adverse drug reaction** | **Cases** | **ROR** | **RORL** | **RORU** | **Pharmacovigilance signal** |
| Obesity subgroup | Female | Anxiety | 83 | 0.56 | 0.44 | 0.72 | N |
|  |  | Depression | 89 | 0.89 | 0.69 | 1.15 | N |
|  |  | Mood disorders | 36 | 0.38 | 0.26 | 0.54 | N |
|  |  | Suicide, and self-harm | 77 | 1.55 | 1.14 | 2.09 | Y |
|  | Male | Anxiety | 23 | 0.86 | 0.53 | 1.4 | N |
|  |  | Depression | 28 | 1.4 | 0.87 | 2.25 | N |
|  |  | Mood disorders | 24 | 1.32 | 0.79 | 2.2 | N |
|  |  | Suicide, and self-harm | 35 | 2.43 | 1.5 | 3.95 | Y |
| Diabetes subgroup | Female | Anxiety | 1929 | 0.99 | 0.94 | 1.04 | N |
|  |  | Depression | 634 | 0.67 | 0.61 | 0.73 | N |
|  |  | Mood disorders | 564 | 0.68 | 0.62 | 0.74 | N |
|  |  | Suicide, and self-harm | 185 | 0.55 | 0.47 | 0.64 | N |
|  | Male | Anxiety | 651 | 0.86 | 0.79 | 0.93 | N |
|  |  | Depression | 330 | 0.73 | 0.65 | 0.82 | N |
|  |  | Mood disorders | 382 | 0.85 | 0.76 | 0.94 | N |
|  |  | Suicide, and self-harm | 156 | 0.63 | 0.54 | 0.75 | N |

| **Table S4. Gender subgroup analysis of risk signals for adverse reactions related to suicide and self-harm in obese patients.** | | | | | | | |
| --- | --- | --- | --- | --- | --- | --- | --- |
| **Drug indications** | **Gender** | **Suicide, and self-harm** | **Cases** | **ROR** | **RORL** | **RORU** | **Pharmacovigilance signal** |
| Obesity subgroup | Female | suicidal ideation | 61 | 2.2 | 1.52 | 3.18 | Y |
|  |  | suicide attempt | 6 | 0.37 | 0.15 | 0.88 | N |
|  |  | completed suicide | 4 | 7.62 | 0.85 | 68.15 | N |
|  |  | self-harm ideation | 3 | 0.95 | 0.24 | 3.81 | N |
|  |  | self-harm behavior. | 3 | 1.43 | 0.32 | 6.38 | N |
|  | Male | suicidal ideation | 25 | 2.15 | 1.23 | 3.75 | Y |
|  |  | suicide attempt | 5 | 2.14 | 0.62 | 7.41 | N |
|  |  | completed suicide | 0 | NA | NA | NA | N |
|  |  | self-harm ideation | 4 | NA | NA | NA | N |
|  |  | self-harm behavior. | 1 | NA | NA | NA | N |

| **Table S5.** **Genetic instrumental variables for GLP1R agonism.** | | | | | | | | | | |
| --- | --- | --- | --- | --- | --- | --- | --- | --- | --- | --- |
| **SNP** | **p** | **effect_allele** | **other_allele** | **N** | **eaf** | **beta** | **se** | **maf** | **r2** | **F** |
| rs1678701 | 3.45E-13 | A | G | 29498 | 0.423519861 | -0.060564106 | 0.008324734 | 0.423519861 | 0.001791096 | 52.92494719 |
| rs1678697 | 4.34E-13 | G | A | 29502 | 0.424569616 | -0.060284394 | 0.008321517 | 0.424569616 | 0.001775749 | 52.47777281 |
| rs1678696 | 4.37E-13 | G | A | 29503 | 0.424553588 | -0.060275366 | 0.008321419 | 0.424553588 | 0.001775199 | 52.46328638 |
| rs1629877 | 4.82E-13 | C | A | 29504 | 0.424650812 | -0.060163726 | 0.008321055 | 0.424650812 | 0.001768736 | 52.27369497 |
| rs1678700 | 5.39E-13 | G | A | 29504 | 0.424273311 | -0.060042816 | 0.008322058 | 0.424273311 | 0.001761222 | 52.05125419 |
| rs1678702 | 5.44E-13 | C | A | 29504 | 0.424405436 | -0.060031221 | 0.00832172 | 0.424405436 | 0.001760686 | 52.03538351 |
| rs1678695 | 5.58E-13 | A | G | 29504 | 0.424613062 | -0.059997466 | 0.008321193 | 0.424613062 | 0.001758932 | 51.98346049 |
| rs1738203 | 6.38E-13 | A | G | 29507 | 0.568338806 | -0.059720399 | 0.008303611 | 0.431661194 | 0.00174995 | 51.722796 |
| rs1678707 | 6.49E-13 | G | A | 29506 | 0.424750868 | -0.059822615 | 0.0083206 | 0.424750868 | 0.001748844 | 51.6882831 |
| rs1678706 | 6.83E-13 | G | A | 29506 | 0.424694247 | -0.059765515 | 0.008320759 | 0.424694247 | 0.001745446 | 51.58768297 |
| rs9380787 | 7.07E-13 | G | T | 29616 | 0.557232539 | -0.059323893 | 0.008264913 | 0.442767461 | 0.001736607 | 51.51733319 |
| rs6919465 | 7.11E-13 | G | A | 29506 | 0.424675374 | -0.059721008 | 0.008320818 | 0.424675374 | 0.001742827 | 51.51014305 |
| rs1678712 | 7.24E-13 | G | A | 29506 | 0.424750868 | -0.059698023 | 0.00832063 | 0.424750868 | 0.001741567 | 51.47283067 |
| rs984524 | 7.43E-13 | G | A | 29506 | 0.424807489 | -0.05966625 | 0.008320492 | 0.424807489 | 0.001739774 | 51.41975483 |
| rs1738199 | 7.61E-13 | A | G | 29505 | 0.565059832 | -0.059468824 | 0.008296548 | 0.434940168 | 0.001738332 | 51.37530751 |
| rs1738212 | 7.80E-13 | C | A | 29506 | 0.424826363 | -0.059611915 | 0.008320457 | 0.424826363 | 0.001736627 | 51.32658112 |
| rs1678693 | 1.04E-12 | C | T | 29506 | 0.425260456 | -0.05927593 | 0.008319429 | 0.425260456 | 0.001717564 | 50.76218381 |
| rs1678691 | 2.06E-12 | C | T | 29506 | 0.426204137 | -0.058473472 | 0.008317233 | 0.426204137 | 0.001672333 | 49.42317317 |
| rs9349100 | 2.36E-12 | T | C | 29507 | 0.563979164 | -0.058150822 | 0.008294226 | 0.436020836 | 0.001663076 | 49.15078964 |
| rs1678692 | 2.50E-12 | C | T | 29508 | 0.426020986 | -0.058249703 | 0.008317465 | 0.426020986 | 0.001659375 | 49.04288694 |
| rs9394544 | 3.05E-12 | A | G | 29500 | 0.569036472 | -0.057943024 | 0.008306648 | 0.430963528 | 0.001646694 | 48.65430143 |
| rs1678682 | 6.45E-12 | G | A | 29508 | 0.565618631 | -0.057000264 | 0.008297948 | 0.434381369 | 0.001596536 | 47.18271035 |

| **Table S6. Mendelian randomization analysis on the causal effect of type 2 diabetes on emotional disorder.** | | | | | | | | | | | | |
| --- | --- | --- | --- | --- | --- | --- | --- | --- | --- | --- | --- | --- |
| **Exposure** | **Outcome** | **Method** | **nsnp** | **beta** | **se** | **P.value** | **OR** | **95% CI_low** | **95% CI_up** | **P.heterogeneity** | **Egger intercept** | **P-intercept** |
| Type 2 diabetes | All anxiety disorders | Inverse variance weighted | 223 | 0.0137 | 0.0146 | 0.3479 | 1.0138 | 0.9852 | 1.0433 | 0.0000 |  |  |
|  |  | MR Egger | 223 | -0.0581 | 0.0305 | 0.0585 | 0.9436 | 0.8888 | 1.0018 | 0.0000 | 0.0049 | 0.0082 |
|  |  | Weighted median | 223 | -0.0132 | 0.0195 | 0.4976 | 0.9869 | 0.9500 | 1.0253 |  |  |  |
|  |  | Simple mode | 223 | 0.0474 | 0.0540 | 0.3816 | 1.0485 | 0.9431 | 1.1657 |  |  |  |
|  |  | Weighted mode | 223 | -0.0266 | 0.0306 | 0.3849 | 0.9737 | 0.9171 | 1.0339 |  |  |  |
|  | Other reaction to severe stress, and adjustment disorders | Inverse variance weighted | 223 | 0.0488 | 0.0160 | 0.0024 | 1.0500 | 1.0175 | 1.0835 | 0.0125 |  |  |
|  |  | MR Egger | 223 | -0.0127 | 0.0337 | 0.7065 | 0.9874 | 0.9243 | 1.0548 | 0.0191 | 0.0042 | 0.0397 |
|  |  | Weighted median | 223 | 0.0291 | 0.0251 | 0.2457 | 1.0296 | 0.9801 | 1.0815 |  |  |  |
|  |  | Simple mode | 223 | -0.0354 | 0.0639 | 0.5802 | 0.9652 | 0.8516 | 1.0940 |  |  |  |
|  |  | Weighted mode | 223 | -0.0153 | 0.0362 | 0.6734 | 0.9848 | 0.9173 | 1.0573 |  |  |  |
|  | Depression | Inverse variance weighted | 223 | 0.0304 | 0.0120 | 0.0115 | 1.0308 | 1.0068 | 1.0554 | 0.0000 |  |  |
|  |  | MR Egger | 223 | -0.0336 | 0.0250 | 0.1814 | 0.9670 | 0.9207 | 1.0156 | 0.0000 | 0.0044 | 0.0041 |
|  |  | Weighted median | 223 | -0.0019 | 0.0156 | 0.9012 | 0.9981 | 0.9681 | 1.0290 |  |  |  |
|  |  | Simple mode | 223 | 0.0064 | 0.0373 | 0.8630 | 1.0065 | 0.9356 | 1.0827 |  |  |  |
|  |  | Weighted mode | 223 | -0.0093 | 0.0214 | 0.6654 | 0.9908 | 0.9501 | 1.0332 |  |  |  |
|  | Emotionally unstable personality disorder | Inverse variance weighted | 223 | 0.0439 | 0.0297 | 0.1396 | 1.0449 | 0.9858 | 1.1076 | 0.0135 |  |  |
|  |  | MR Egger | 223 | -0.0568 | 0.0625 | 0.3650 | 0.9448 | 0.8359 | 1.0680 | 0.0184 | 0.0069 | 0.0691 |
|  |  | Weighted median | 223 | 0.0555 | 0.0491 | 0.2583 | 1.0570 | 0.9601 | 1.1637 |  |  |  |
|  |  | Simple mode | 223 | 0.0704 | 0.1102 | 0.5233 | 1.0729 | 0.8646 | 1.3315 |  |  |  |
|  |  | Weighted mode | 223 | 0.0625 | 0.0647 | 0.3348 | 1.0645 | 0.9377 | 1.2085 |  |  |  |
|  | Bipolar affective disorders | Inverse variance weighted | 223 | 0.0103 | 0.0245 | 0.6739 | 1.0104 | 0.9629 | 1.0602 | 0.0037 |  |  |
|  |  | MR Egger | 223 | -0.0716 | 0.0518 | 0.1679 | 0.9309 | 0.8411 | 1.0303 | 0.0052 | 0.0056 | 0.0740 |
|  |  | Weighted median | 223 | 0.0297 | 0.0366 | 0.4172 | 1.0302 | 0.9588 | 1.1069 |  |  |  |
|  |  | Simple mode | 223 | 0.0542 | 0.0933 | 0.5617 | 1.0557 | 0.8793 | 1.2676 |  |  |  |
|  |  | Weighted mode | 223 | 0.0475 | 0.0771 | 0.5387 | 1.0486 | 0.9015 | 1.2197 |  |  |  |
|  | Suicide or other Intentional self-harm | Inverse variance weighted | 223 | 0.0336 | 0.0198 | 0.0886 | 1.0342 | 0.9949 | 1.0750 | 0.0742 |  |  |
|  |  | MR Egger | 223 | -0.0254 | 0.0417 | 0.5434 | 0.9749 | 0.8984 | 1.0580 | 0.0862 | 0.0040 | 0.1100 |
|  |  | Weighted median | 223 | -0.0059 | 0.0305 | 0.8457 | 0.9941 | 0.9363 | 1.0554 |  |  |  |
|  |  | Simple mode | 223 | 0.0627 | 0.0716 | 0.3819 | 1.0647 | 0.9253 | 1.2252 |  |  |  |
|  |  | Weighted mode | 223 | -0.0120 | 0.0390 | 0.7589 | 0.9881 | 0.9153 | 1.0666 |  |  |  |

| **Table S7.** **Mendelian randomization analysis on the causal effect of obesity on emotional disorder.** | | | | | | | | | | | | |
| --- | --- | --- | --- | --- | --- | --- | --- | --- | --- | --- | --- | --- |
| **Exposure** | **Outcome** | **Method** | **nsnp** | **beta** | **se** | **P.value** | **OR** | **95% CI_low** | **95% CI_up** | **P.heterogeneity** | **Egger intercept** | **P-intercept** |
| Obesity | All anxiety disorders | Inverse variance weighted | 59 | 0.0291 | 0.0281 | 0.2994 | 1.0296 | 0.9744 | 1.0878 | 0.0000 |  |  |
|  |  | MR Egger | 59 | -0.0988 | 0.0713 | 0.1711 | 0.9059 | 0.7877 | 1.0418 | 0.0000 | 0.0097 | 0.0567 |
|  |  | Weighted median | 59 | -0.0385 | 0.0309 | 0.2135 | 0.9622 | 0.9056 | 1.0224 |  |  |  |
|  |  | Simple mode | 59 | -0.0559 | 0.0576 | 0.3360 | 0.9457 | 0.8447 | 1.0587 |  |  |  |
|  |  | Weighted mode | 59 | -0.0526 | 0.0359 | 0.1481 | 0.9487 | 0.8843 | 1.0179 |  |  |  |
|  | Other reaction to severe stress, and adjustment disorders | Inverse variance weighted | 59 | 0.0454 | 0.0270 | 0.0924 | 1.0464 | 0.9926 | 1.1033 | 0.0278 |  |  |
|  |  | MR Egger | 59 | 0.0368 | 0.0708 | 0.6049 | 1.0375 | 0.9032 | 1.1918 | 0.0227 | 0.0007 | 0.8959 |
|  |  | Weighted median | 59 | -0.0015 | 0.0387 | 0.9688 | 0.9985 | 0.9256 | 1.0771 |  |  |  |
|  |  | Simple mode | 59 | -0.0447 | 0.0628 | 0.4795 | 0.9563 | 0.8456 | 1.0815 |  |  |  |
|  |  | Weighted mode | 59 | -0.0197 | 0.0456 | 0.6681 | 0.9805 | 0.8967 | 1.0722 |  |  |  |
|  | Depression | Inverse variance weighted | 59 | 0.0651 | 0.0234 | 0.0055 | 1.0673 | 1.0193 | 1.1175 | 0.0000 |  |  |
|  |  | MR Egger | 59 | 0.0263 | 0.0612 | 0.6694 | 1.0266 | 0.9106 | 1.1575 | 0.0000 | 0.0029 | 0.4946 |
|  |  | Weighted median | 59 | 0.0487 | 0.0221 | 0.0271 | 1.0499 | 1.0055 | 1.0963 |  |  |  |
|  |  | Simple mode | 59 | 0.0026 | 0.0459 | 0.9554 | 1.0026 | 0.9163 | 1.0970 |  |  |  |
|  |  | Weighted mode | 59 | 0.0730 | 0.0319 | 0.0256 | 1.0758 | 1.0106 | 1.1452 |  |  |  |
|  | Emotionally unstable personality disorder | Inverse variance weighted | 59 | 0.1564 | 0.0426 | 0.0002 | 1.1693 | 1.0757 | 1.2710 | 0.4898 |  |  |
|  |  | MR Egger | 59 | -0.0448 | 0.1106 | 0.6871 | 0.9562 | 0.7698 | 1.1877 | 0.5987 | 0.0153 | 0.0536 |
|  |  | Weighted median | 59 | 0.1339 | 0.0667 | 0.0448 | 1.1433 | 1.0031 | 1.3030 |  |  |  |
|  |  | Simple mode | 59 | 0.2163 | 0.1433 | 0.1366 | 1.2415 | 0.9375 | 1.6441 |  |  |  |
|  |  | Weighted mode | 59 | 0.0805 | 0.0942 | 0.3967 | 1.0838 | 0.9010 | 1.3036 |  |  |  |
|  | Bipolar affective disorders | Inverse variance weighted | 59 | 0.0850 | 0.0457 | 0.0626 | 1.0888 | 0.9955 | 1.1907 | 0.0003 |  |  |
|  |  | MR Egger | 59 | 0.1716 | 0.1191 | 0.1550 | 1.1872 | 0.9401 | 1.4994 | 0.0003 | 0.0083 | 0.4341 |
|  |  | Weighted median | 59 | 0.0773 | 0.0606 | 0.2016 | 1.0804 | 0.9595 | 1.2166 |  |  |  |
|  |  | Simple mode | 59 | 0.1549 | 0.1246 | 0.2187 | 1.1675 | 0.9146 | 1.4903 |  |  |  |
|  |  | Weighted mode | 59 | 0.0775 | 0.0704 | 0.2758 | 1.0805 | 0.9413 | 1.2404 |  |  |  |
|  | Suicide or other Intentional self-harm | Inverse variance weighted | 59 | 0.0437 | 0.0386 | 0.2576 | 1.0446 | 0.9686 | 1.1267 | 0.0004 |  |  |
|  |  | MR Egger | 59 | 0.0719 | 0.1011 | 0.4795 | 1.0746 | 0.8815 | 1.3100 | 0.0003 | -0.0021 | 0.7629 |
|  |  | Weighted median | 59 | 0.0224 | 0.0515 | 0.6635 | 1.0227 | 0.9245 | 1.1313 |  |  |  |
|  |  | Simple mode | 59 | 0.0327 | 0.1048 | 0.7562 | 1.0332 | 0.8414 | 1.2689 |  |  |  |
|  |  | Weighted mode | 59 | 0.0284 | 0.0614 | 0.6451 | 1.0289 | 0.9121 | 1.1605 |  |  |  |

| **Table S8.** **Steiger Analysis for Causal Directionality Validation** | | | | | |
| --- | --- | --- | --- | --- | --- |
| **exposure** | **outcome** | **snp_r2.exposure** | **snp_r2.outcome** | **correct_causal_direction** | **steiger_pval** |
| GLP-1RA | Type 2 diabetes | 0.038068333 | 6.18E-05 | TRUE | 7.05E-220 |
| GLP-1RA | Obesity | 0.038068333 | 0.000345463 | TRUE | 2.93E-196 |
| GLP-1RA | All anxiety disorders | 0.038068333 | 0.000328119 | TRUE | 7.76E-197 |
| GLP-1RA | Stress | 0.038068333 | 3.56E-05 | TRUE | 1.30E-223 |
| GLP-1RA | Depression | 0.038068333 | 4.51E-05 | TRUE | 1.04E-222 |
| GLP-1RA | Bipolar affective disorders | 0.038068333 | 0.000365837 | TRUE | 9.87E-194 |
| GLP-1RA | Emotionally unstable personality disorder | 0.038068333 | 0.001460288 | TRUE | 7.65E-156 |
| GLP-1RA | Suicide or other Intentional self-harm | 0.038068333 | 0.000118186 | TRUE | 2.32E-213 |

| **Table S9. Summary-data-based Mendelian randomization analysis on the causal effect of GLP1R on emotional disorder.** | | | | | | | | | | |
| --- | --- | --- | --- | --- | --- | --- | --- | --- | --- | --- |
| **Outcome** | **probeID** | **Chr** | **Gene** | **Probe_bp** | **topSNP** | **topSNP_chr** | **topSNP_bp** | **A1** | **A2** | **Freq** |
| All anxiety disorders | ENSG00000112164 | 6 | GLP1R | 39036046 | rs9283907 | 6 | 39026703 | A | G | 0.170974 |
| stress | ENSG00000112164 | 6 | GLP1R | 39036046 | rs9283907 | 6 | 39026703 | A | G | 0.170974 |
| Depression | ENSG00000112164 | 6 | GLP1R | 39036046 | rs9283907 | 6 | 39026703 | A | G | 0.170974 |
| Emotionally unstable personality disorder | ENSG00000112164 | 6 | GLP1R | 39036046 | rs9283907 | 6 | 39026703 | A | G | 0.170974 |
| Bipolar affective disorders | ENSG00000112164 | 6 | GLP1R | 39036046 | rs9283907 | 6 | 39026703 | A | G | 0.170974 |
| Suicide or other Intentional self-harm | ENSG00000112164 | 6 | GLP1R | 39036046 | rs9283907 | 6 | 39026703 | A | G | 0.170974 |

| **Table S9. Summary-data-based Mendelian randomization analysis on the causal effect of GLP1R on emotional disorder. (Continued)** | | | | | | | | | | | |
| --- | --- | --- | --- | --- | --- | --- | --- | --- | --- | --- | --- |
| **Outcome** | **b_GWAS** | **se_GWAS** | **p_GWAS** | **b_eQTL** | **se_eQTL** | **p_eQTL** | **b_SMR** | **se_SMR** | **p_SMR** | **p_HEIDI** | **nsnp_HEIDI** |
| All anxiety disorders | -0.0240 | 0.0108 | 0.0261 | 0.1013 | 0.0115 | 0.0000 | -0.2371 | 0.1099 | 0.0310 | 0.3370 | 19 |
| stress | 0.0045 | 0.0143 | 0.7530 | 0.1013 | 0.0115 | 0.0000 | 0.0443 | 0.1412 | 0.7540 | 0.1620 | 19 |
| Depression | -0.0099 | 0.0086 | 0.2480 | 0.1013 | 0.0115 | 0.0000 | -0.0976 | 0.0852 | 0.2520 | 0.6290 | 19 |
| Emotionally unstable personality disorder | -0.0059 | 0.0266 | 0.8240 | 0.1013 | 0.0115 | 0.0000 | -0.0584 | 0.2623 | 0.8240 | 0.6870 | 19 |
| Bipolar affective disorders | -0.0138 | 0.0215 | 0.5210 | 0.1013 | 0.0115 | 0.0000 | -0.1361 | 0.2126 | 0.5220 | 0.3670 | 19 |
| Suicide or other Intentional self-harm | 0.0127 | 0.0183 | 0.4880 | 0.1013 | 0.0115 | 0.0000 | 0.1250 | 0.1807 | 0.4890 | 0.3190 | 19 |
